# Toward a Circular Bioeconomy: Exploring Pineapple Stem Starch Film as a Plastic Substitute in Single Use Applications

**DOI:** 10.3390/membranes13050458

**Published:** 2023-04-24

**Authors:** Atitiya Namphonsane, Phattarakarn Suwannachat, Chin Hua Chia, Rungtiwa Wongsagonsup, Siwaporn Meejoo Smith, Taweechai Amornsakchai

**Affiliations:** 1 Center of Sustainable Energy and Green Materials, Faculty of Science, Mahidol University, Phuttamonthon 4 Road, Nakhon Pathom 73170, Thailandsiwaporn.smi@mahidol.ac.th (S.M.S.); 2 Department of Applied Physics, Faculty of Science and Technology, Universiti Kebangsaan Malaysia, Bangi 43600, Selangor, Malaysia; chia@ukm.edu.my; 3 Division of Food Technology, Kanchanaburi Campus, Mahidol University, Kanchanaburi 71150, Thailand; rungtiwa.won@mahidol.ac.th

**Keywords:** agricultural waste, pineapple stem, starch, amylose, film

## Abstract

In this study, biodegradable starch film was developed from pineapple stem waste as a substitute for non-biodegradable petroleum-based films for single-use applications where strength is not too demanding. High amylose starch from a pineapple stem was used as the matrix. Glycerol and citric acid were used as additives to adjust the ductility of the material. Glycerol content was fixed at 25% while that of citric acid varied from 0 to 15% by weight of starch. Films with a wide range of mechanical properties can be prepared. As more citric acid is added, the film becomes softer and weaker, and has greater elongation at the break. Properties range from a strength of about 21.5 MPa and 2.9% elongation to a strength of about 6.8 MPa and 35.7% elongation. An X-ray diffraction study showed that the films were semi-crystalline. The films were also found to be water-resistant and can be heat-sealed. An example of a single-use package was demonstrated. A soil burial test confirmed that the material was biodegradable and completely disintegrated into sizes smaller than 1 mm within one month.

## 1. Introduction

Plastics are used in almost every aspect of our daily lives and new uses are found regularly. This is because they are lightweight and can be turned into products easily and economically. Although plastic parts can be collected and recycled, those with a very thin gauge or which are very small in size are likely to be ignored and thus escape the cycle into the environment, posing different kinds of notorious problems. The recent COVID-19 pandemic has exacerbated plastic waste problems due to the increased use of protective personal equipment and single-use plastic packaging [1].

To tackle the aforementioned problems, both synthetic and natural biodegradable polymers were studied and developed. For the former category, polylactic acid (PLA) is probably the best-known and most studied polymer, followed by polybutylene succinate (PBS) and polybutylene adipate terephthalate (PBAT). The latter includes starch, polyhydroxyalkanoates (PHAs) and polyhydroxybutyrate (PHB). However, PLA, PBAT and PBS are not biodegraded easily in natural environments but in industrial compost facilities under a controlled humidity and temperature [2,3]. Only starch and PHAs and PHB are fully biodegradable in natural environments.

Starch has received a great deal of attention because it is widely available at a very low cost and there are many reviews available; see, for example, [4,5,6,7,8]. However, starch has poor properties, especially low mechanical strength, and low water resistance, and requires modification or blending with other polymers [9,10,11,12,13]. More importantly, most starches are for human consumption and using them for materials would certainly interrupt food supply chains and could limit vulnerable groups to food access and hence is not very sustainable. Various non-conventional or non-food starches have been researched [14,15,16,17,18,19,20]. The availability of these non-conventional starches is relatively limited. So far, most work on starch for material applications has been devoted to modification to overcome its poor water-resistant and low mechanical properties. Starch modification started with a single step method and yet the obtained products still had limitations; now, modification has advanced to dual modifications [21,22,23,24] or combined with various fillers, modifiers or even film-forming polymers [25,26,27]. This not only complicates the process but also results in products with greater material and energy intensities as well as a larger carbon footprint. Therefore, finding a starch that requires little or no modification would be more sustainable. If the starch can be derived from waste biomass, this would lead to even more sustainability.

Pineapple production is one of the most important agricultural industries. There are different kinds of residues associated with the industry, such as peels, crowns, leaves, and stems. The latter three are left in the field for farmers to handle before a new crop cultivation cycle can be started. These residues are under-utilized and need more attention, especially in the current situation due to the complex problems mentioned above. In certain parts of Thailand, the pineapple stem is used as a source of bromelain extraction [28] and the residue is used as animal feed; information on the amount of this is not publicly available. The pineapple stem is known to contain starch with high amylose content [29,30]. Hereafter, the starch is denoted as PSS. Only recently has more attention been paid to exploring different ways to utilize this PSS [31,32,33,34,35,36]. To the best of our knowledge, there is very limited work on the use of PSS as packaging material. If PSS can be used for material production, a circular economy in the industry could be better realized.

In this paper, we explore the development of water-resistant biodegradable starch films using unmodified PSS for single-use applications. The matrix consisted of raw starch from the pineapple stem. Glycerol and citric acid were used as property modifiers to expand the range of the film’s mechanical properties [6]. Glycerol was chosen primarily because it is the most widely used plasticizer while citric acid can act as both a cross-linking agent and as a plasticizer [6,37,38,39,40,41]. In addition, these additives were chosen due to their biobased nature and low cost, and are readily available in Thailand. The focus of the research was on the range of mechanical properties of the films and their water resistance in terms of water absorption and solubility. The final part demonstrates a simple protective package made from PSS film and evaluates the degradation of the film in a natural environment. This work has practical implications for the current era of the pandemic, which has necessitated protective packaging while causing a surge in plastic waste.

## 2. Materials and Methods

### 2.1. Materials

Pineapple stem waste, a byproduct of a proprietary bromelain extraction process, was obtained from local source (Hong Mao Biochem, Rayong, Thailand). In general, the process involves crushing peeled pineapple stems to disrupt the cell structure and liquid extracted by centrifugation [28]. The remaining solid material was dried under the sun for a few days and further ground into powder using a grinder. The stem powder was collected by sieving (80 mesh) to separate the coarse fibers, cell wall and other solid contaminants which constitute about 56% of the whole mass. The powder was used as obtained without further washing. The number of extractive constituents makes up about 15% of the dry powder. The characteristic of the powder is similar to that obtained by the wet milling reported previously [30]. Glycerol and citric acid are commercial grade and obtained from local stores.

### 2.2. Preparation of Starch Paste and Films

Starch paste was prepared by mixing PSS powder and water in a 1:10 weight ratio. Different amounts of glycerol and citric acid were added to the mixture. The amount of the glycerol was fixed at 25% by weight of starch while that of the citric acid was 0, 5, 10 and 15%. The samples are coded as G25CA0, G25CA5, G25CA10 and G25CA15, respectively. The mixture was left for at least 20 min before being gelatinized in a household microwave (Toshiba, model ER-G33SC(S)) set at 30% of maximum power (1100 kW) for 30 s three times. When the citric acid was added to the mixture, it was necessary to increase the level of microwave heating until the whole mixture became gelatinized. The gelatinized PSS was further homogenized by continuous stirring at 70 °C and 250 rpm for 1 h. After that, it was left to cool down to room temperature before being cast in a silicone mold. The samples were air-dried at room temperature to form homogeneous films.

### 2.3. Characterization of Pineapple Stem Starch Films

FTIR. FTIR spectra of the films were obtained by measuring them in attenuated total reflection (ATR) mode on a spectrophotometer (Frontier, Perkin Elmer, Waltham, MA, USA). The measurements were performed at room temperature over a range of 4000 to 400 cm^−1^ with a resolution of 4 cm^−1^.

XRD. X-ray diffraction patterns of the films were obtained from a benchtop X-ray powder diffractometer (D2 Phaser, Bruker, Germany) using an X-ray wavelength of 1.54 Å with a step scan of 15 s/point over the 2θ of 5–40 degrees. The percentage crystallinity of each PSS film sample was determined using Equation (1) [21].

Crystallinity (%) = A_c_/(A_c_ + A_a_) × 100 (1)

A_c_ = the area of the crystallinity region and A_a_ = the area of the amorphous region

Mechanical properties. Specimens were cut from PSS films into strips of 6 mm wide and 110 mm long using a sharp blade. Tensile tests were performed following [21] with some modification on a universal testing machine (Instron 5569, High Wycombe, UK) with a long travel contact type extensometer. A gauge length and crosshead speed of 100 mm and 50 mm/min, respectively, were used. A secant modulus at 1%, tensile strength and an elongation at the break were average values from five specimens.

Morphology. Surfaces and fractured surfaces of PSS films were observed with a scanning electron microscope (SEM) (JSM-IT500, JEOL, Tokyo, Japan). The samples were coated with platinum before the observation.

Contact angle, water resistance, solubility and absorption. The static water contact angle was determined using a sessile drop method on a Kruss G-1 contact angle goniometer (Kruss GmbH, Hamburg, Germany) at an ambient temperature. Each contact angle reported in this work was an average of the values obtained for ten points on the sample surfaces. For the water resistance, solubility and absorption, a piece of PSS film was immersed in distilled water for 24 h and its weights (wet and dried) were monitored [21,24]. It should be noted that “water resistant” here describes the ability of the film to resist (liquid) water penetration. Thus, it would be determined qualitatively by observing the uptake of water by the film samples [42]. Water solubility and absorption of the films were determined using Equations (2) and (3), respectively.

Water solubility = ((w_i_ − w_fd_)/w_i_) × 100 (2)

Water absorption = ((w_f_ − w_i_)/w_i_) × 100 (3)

where:

w_fd_ is the weight of the dried PSS film after being immersed in distilled water.

w_f_ is the weight of the wet PSS film after being immersed in distilled water.

w_i_ is the initial weight of the PSS film.

Heat-seal strength test. The test followed the method used in [43] with some modifications. Two pieces of the film samples with a dimension of approximately 5 × 10 cm^2^ were heat-sealed at one end using a locally made commercial heat sealer. The sealing condition was 110–120 °C with approximately 5 kg of force for 10 s. Specimens of about 2.5-cm width and 10-cm length were then cut, and the seal strength was determined by pulling the free ends apart in a universal testing machine (Instron 5569) at a speed of 5 mm/min. The seal strength is reported as the average pulling force divided by the sample width (N/m) [43].

Soil burial test. This test can be used to determine the biodegradability of starch-based films by microorganisms [37,42]. The test was slightly modified from [37]. Film samples of size 4.0 × 4.0 cm^2^ were put in envelopes made from a high-density polyethylene net for easy recovery. The envelopes were buried in the edge of the garden of the department building about 10 cm beneath the surface. The pH of the soil was measured to be 7.5. The area was under the shade of trees and was watered every week. No attempt was made to regulate the moisture content and temperature of the area to obtain a natural environment. Envelopes were taken out for the observation of the sample after different periods of time. The state of biodegradability was evaluated visually after brushing off the soil.

## 3. Results

### 3.1. Film Formation Ability and Quality

**Figure 1:**
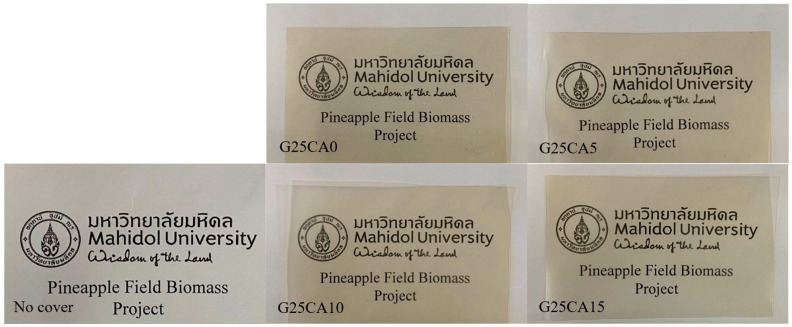
The appearance of PSS films prepared with 25% glycerol and different amounts of citric acid.

All paste compositions were successfully cast into films. However, those without glycerol and with a glycerol content less than 25% were too fragile to handle and not suitable for packaging film applications. Therefore, no further investigation was conducted on these film samples. Photographs of the PSS films prepared with glycerol content of 25% and citric acid of 0, 5, 10 and 15% are shown in Figure 1. All films are slightly yellowish. It was observed that the PSS film without citric acid tends to shrink unevenly (not shown), and the shrinkage becomes greater even as the citric acid content is increased.

### 3.2. FTIR

**Figure 2:**
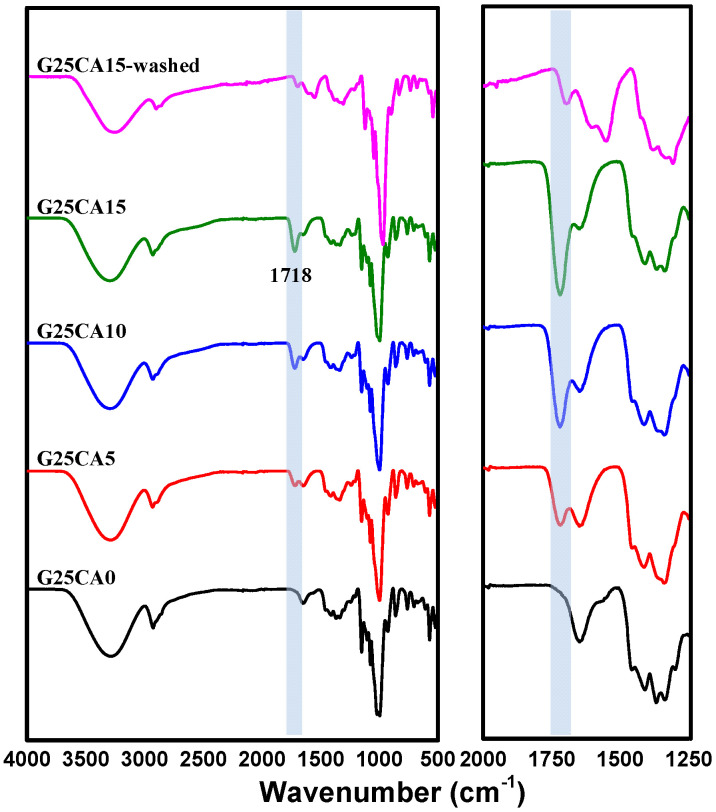
Infrared spectra of PSS films prepared with 25% glycerol and different amounts of citric acid. The shaded region of the figure represents the most obvious change in the spectra. The expanded region is shown on the right.

Infrared spectra of the PSS films are shown in Figure 2. The spectrum of the PSS film with a glycerol content of 25% is shown on top. When citric acid (5%) was added to the system, a new peak appeared at about 1717 cm^−1^. With the increasing citric acid content, the peak becomes stronger and shifts slightly to a higher wavenumber of 1718 cm^−1^ for both the citric acid of 10 and 15%. The slight change in peak position is due to the overlapping nature of the nearby peak. This peak is due to the carboxyl or ester carbonyl functional group [38,39]. Because citric acid has three carboxylic groups, it is likely that esterification would take place to a certain degree. The esterification includes monoester in which citric acid is grafted onto the starch chain and diester in which the citric acid acts as a crosslinking agent between starch chains [40,41]. There could also be free citric acid that did not react with the starch. An additional experiment was conducted by precipitating the starch solution in ethanol to wash away the unreacted citric acid and it was found that the intensity of the 1718 cm^−1^ peak dropped sharply, as shown in Figure 2.

### 3.3. Crystalline Structure

**Figure 3:**
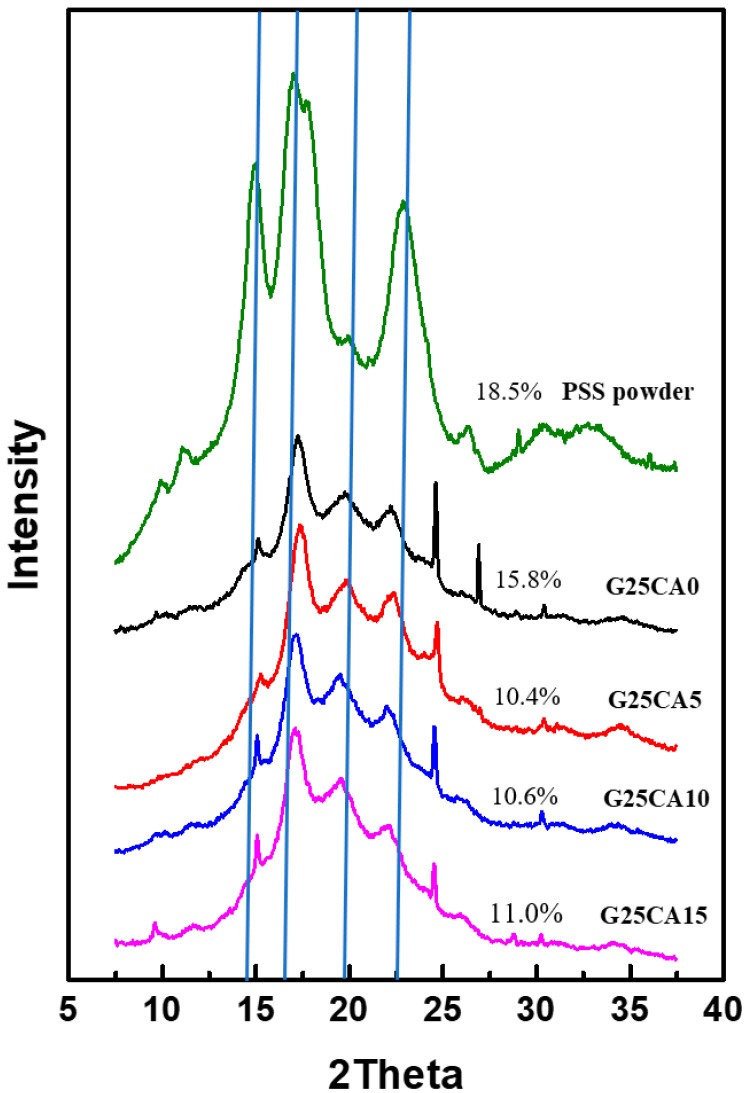
XRD patterns of PSS films prepared with 25% glycerol and different amounts of citric acid. A pattern of PSS powder was added for comparison. The crystallinity index (CI) for each sample is shown by numbers next to the patterns. Vertical lines have been drawn to aid the eye with the relative peak positions.

X-ray diffraction patterns of starting PSS powder and PSS films are shown in Figure 3. PSS displays A-type crystallinity similar to other types of starches as shown previously [30]. The crystallinity of the starting PSS is about 18.5%. After the PSS was gelatinized and dried, all PSS films displayed a similar diffraction pattern which is different from the starting PSS powder. The appearance is similar to that of ozonated cassava starch films [13]. These crystalline peaks are attributed to the spontaneous recrystallization of amylose molecules during film drying [44,45] or retrogradation, and it was stated that retrograded starch is always B-type regardless of the starch type [13,46]. The crystallinity of the PSS film with 25% glycerol is about 15.8% and decreases to about 10.5–11.0% with further addition of citric acid. It should be noted that crystalline scatterings observed are much stronger than those of the cassava starch film reported elsewhere [21,22], suggesting a greater extent of retrogradation. The new patterns are very similar in appearance to that of the pea starch film [47], which is high in amylose content but quite different from that of high amylose corn starch [40]. This is presumably due to the difference in processing method (melt processing) in the latter case. When compared with PSS powder, there are extra peaks at approximately 17.2° and 22.3° which are characteristic of B-type structure forms in the retrogradation process [48,49,50,51,52]. A peak at about 19.8° which associates with the V-type is also present [47,50,53]. XRD that consists of a mixture of B- and V-type structures has been previously reported [48,49,50]. It should be noted that most thermoplastic starches reported do not display such a distinct crystalline structure as is observed here [22,27]. In addition, there are a few sharp peaks which appear at about 15.05°, 24.5° and 26.8° and deserve mentioning. These peaks are not normally observed for retrograded starch except in one study which inferred that crystal with a well-defined morphology had formed [54] after several retrogradation–hydrolysis cycles. However, it is not clear how these peaks originated in our work. Further study will be required to answer this question, and this will be dealt with in future communications.

### 3.4. Mechanical Properties

**Figure 4:**
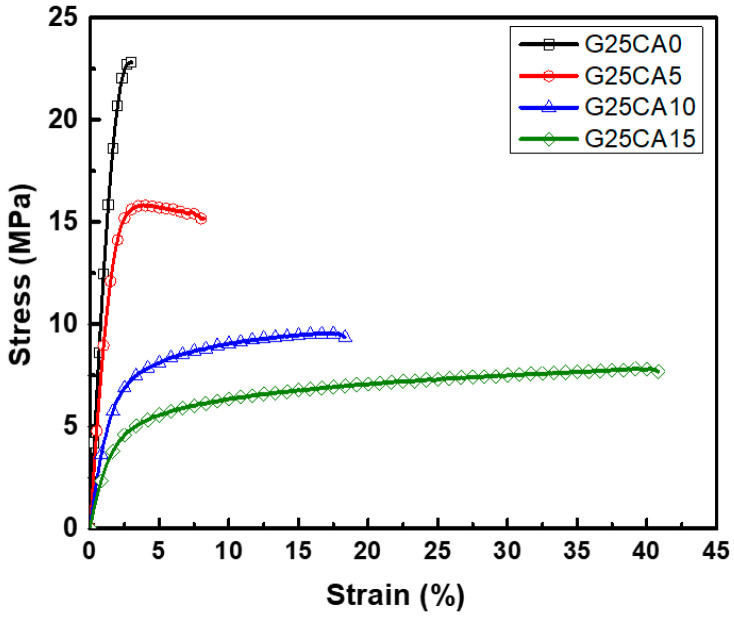
Representative stress–strain curves of PSS films prepared with 25% glycerol and different amounts of citric acid.

**Figure 5:**
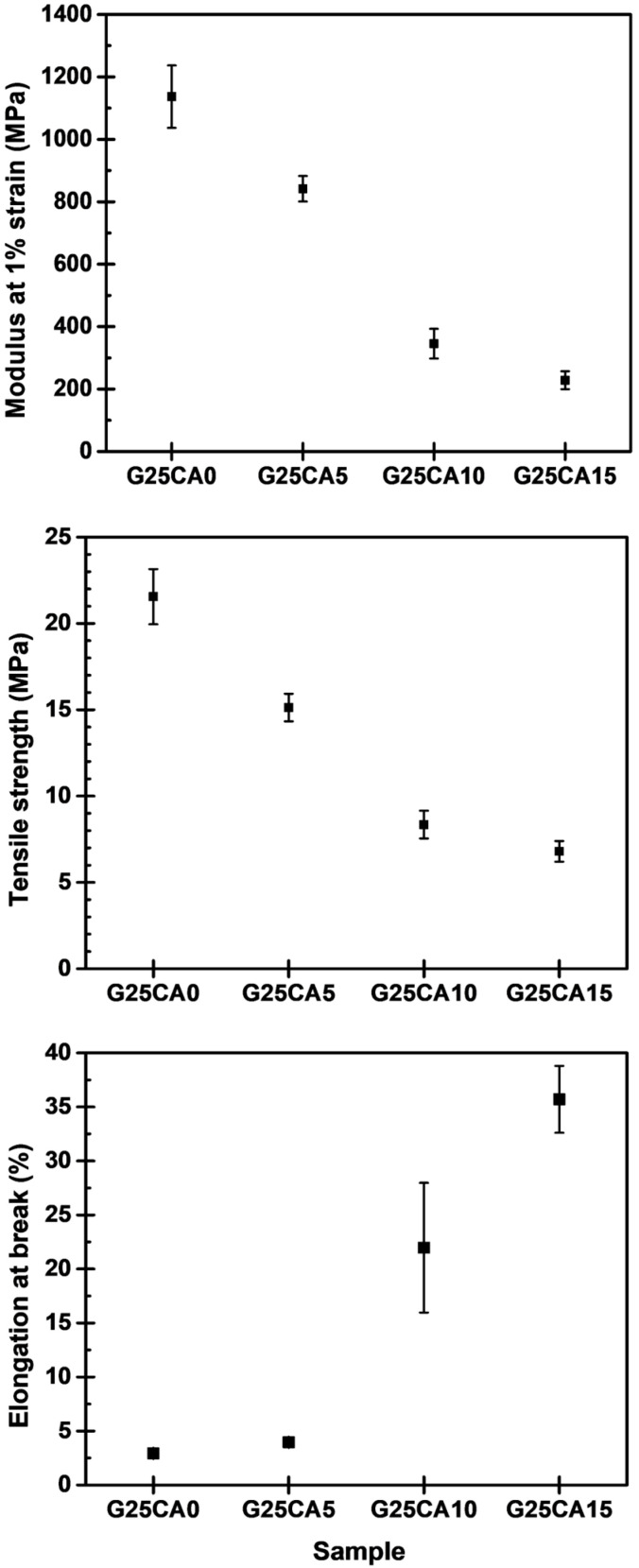
Mechanical properties of PSS films prepared with 25% glycerol and different amounts of citric acid.

Stress–strain curves of different PSS films are shown in Figure 4. A film without citric acid (G25CA0) displays the highest tensile strength and the lowest elongation at the break. As the amount of citric acid increases, the tensile strength decreases while the elongation at the break increases at a similar rate to that observed in the corn starch-based system [55]. This fact indicates that citric acid also acts as a plasticizer for the system. The range of obtainable properties is relatively wide. Figure 5 displays the average tensile strength, modulus, and elongation at the break of the PSS films. The tensile strength is in the range of about 21.6–6.8 MPa and the modulus 1140–230 MPa. The greatest strength obtained is significantly greater than that of other starch-based material containing the same amount of glycerol [13,21,22,55]. The mechanical properties of these PSS films, except for the elongation at the break, fall in the range of low-density polyethylene film [56].

### 3.5. Morphology

**Figure 6:**
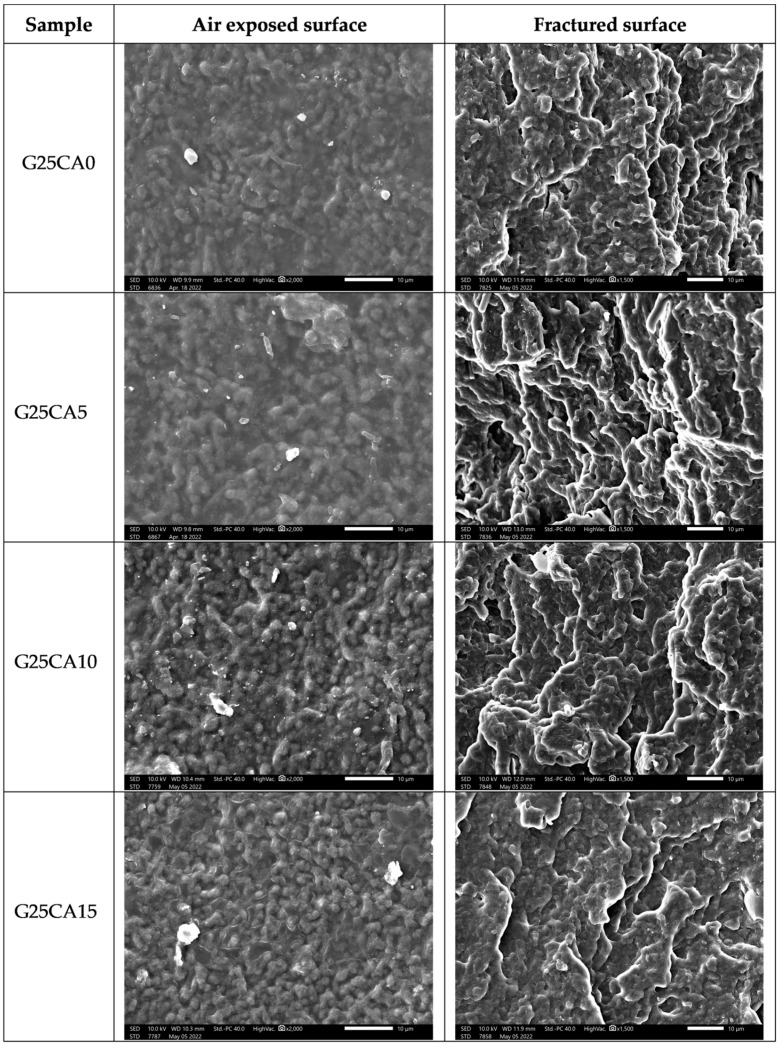
SEM images of PSS films prepared with 25% glycerol and different amounts of citric acid; air exposed surface (**left**) and fractured surface (**right**).

A scanning electron micrograph of the film surfaces and the internal structure of broken films containing 25% glycerol and different amount of citric acid are shown in Figure 6. The film surfaces and the internal structure of the film display a granular structure similar to that reported by Liu [57] for high-amylose films. The size of the granules is about 1–2 µm. This size is much smaller than that of the granular size of the PSS which is about 10 µm [30]. Thus, it could be deduced that this granular structure forms during the retrogradation process. Further investigation regarding the nature of the structure is underway and will be reported in the future.

### 3.6. Contact Angle, Water Solubility and Absorption

**Figure 7:**
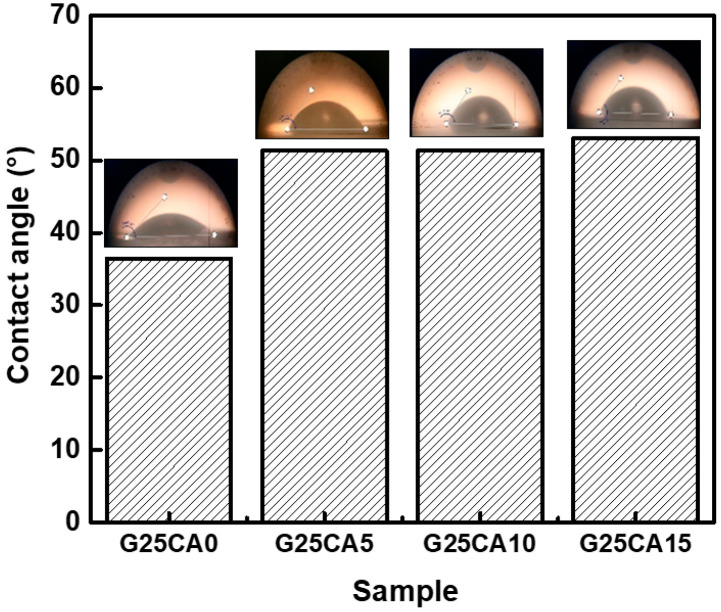
Water contact angle of PSS films prepared with 25% glycerol and different amounts of citric acid.

**Figure 8:**
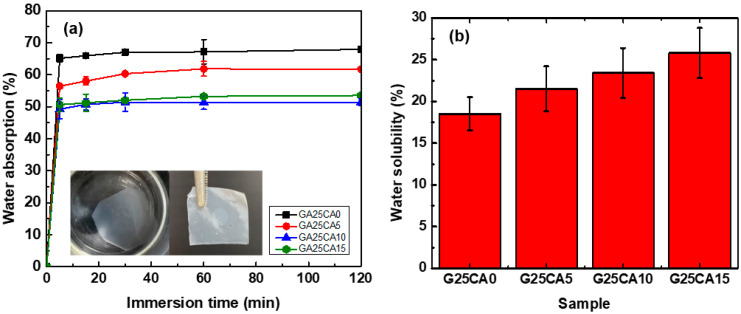
(**a**) Water absorption; insert is a demonstration of water resistivity of the PSS film where a piece of G25CA15 film was immersed in distilled water for 24 h and taken out to dry and (**b**) water solubility of different PSS films.

Water contact angles of the PSS films are shown in Figure 7. A film without citric acid has a contact angle of 36° and when citric acid was introduced, the contact angle increased to 51°, 51° and 53° for citric acid contents of 5%, 10% and 15%, respectively. Water contact angles were less than 90 degrees, suggesting that all PSS film surfaces are favorable for wetting. This is not surprising as the starch molecular structure itself contains a large number of hydroxyl groups. However, no change in the surface appearance of the films after the test was observed, indicating good water-resistant characteristics of high-amylose PSS film [44]. Figure 8 displays the water absorption and solubility of PSS films. By immersing the PSS films in distilled water for different periods of time, the films gain some weight due to water uptake. It appears that water uptake occurs and reaches plateau values within quite a short time due to their thinness. All films retained their shapes with a slight swelling and did not disintegrate into small pieces throughout the entire experiment, indicating good water resistivity compared to, for example, cassava starch-based film [42]. The absorption is slightly lower than 70% for G25CA0 and as the amount of citric acid increases, the water absorption decreases. This could be due to the tighter network of crosslinked structures suggesting that citric acid also acts as a crosslinking agent [39,58]. It should be noted that these PSS films display much lower water absorption than that of other starch films which are in the range of 200–400% [19,20]. The absorption is close to that of high-amylose mung bean crosslinked with 30% malic acid [59]. On the other hand, water solubility of the films increases with the increasing amount of citric acid. This fact would suggest that some citric acids are free and unbound to starch molecules as already shown in the FTIR section.

At first sight, the results for the contact angle and water absorption may seem contradictory, i.e., no difference in the contact angle was observed between G25CA5 and GA25CA10, while there is a clear difference between the water absorption of the two samples. The water contact angle is measured within seconds, compared to the longer time frame of the water absorption. The addition of citric acid affects the contact angle immediately but not during prolonged submersion. When we prolonged the submersion time, the sample with the hydrophobic surface could not resist water penetration. Despite the difference in water absorption between the samples, all films remained intact with some slight swelling and did not disintegrate into smaller pieces.

### 3.7. Heat Sealability and Strength

**Figure 9:**
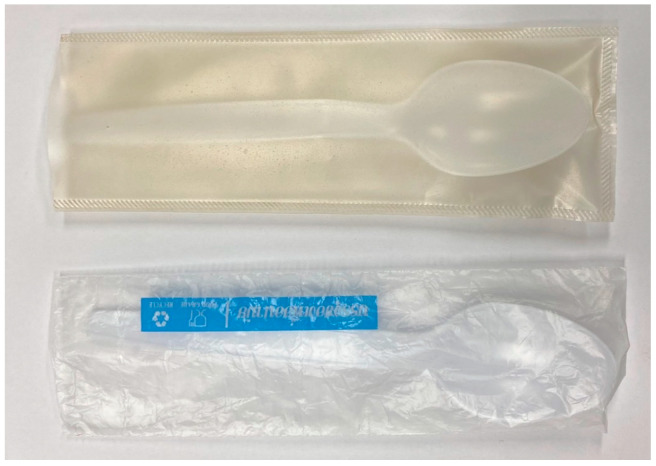
Transparent protective envelope made from G25CA15 film (**top**) compared with that made from HDPE film (**bottom**).

Heat sealings of PSS films were performed in order to demonstrate their ability to be transformed into different packaging products. It was found that films without citric acid (G25CA0) did not stick to each other. Only films with good ductility (G25CA10 and G25CA15) can be heat-sealed and their seal strengths were 253.2 and 393.6 N/m, respectively. The films can be easily transformed into a package with a heat sealer. An example of a transparent protective envelope made from G25CA10 film is shown in Figure 9 along with a commercial envelope made from HDPE.

### 3.8. Soil Burial Test

**Figure 10:**
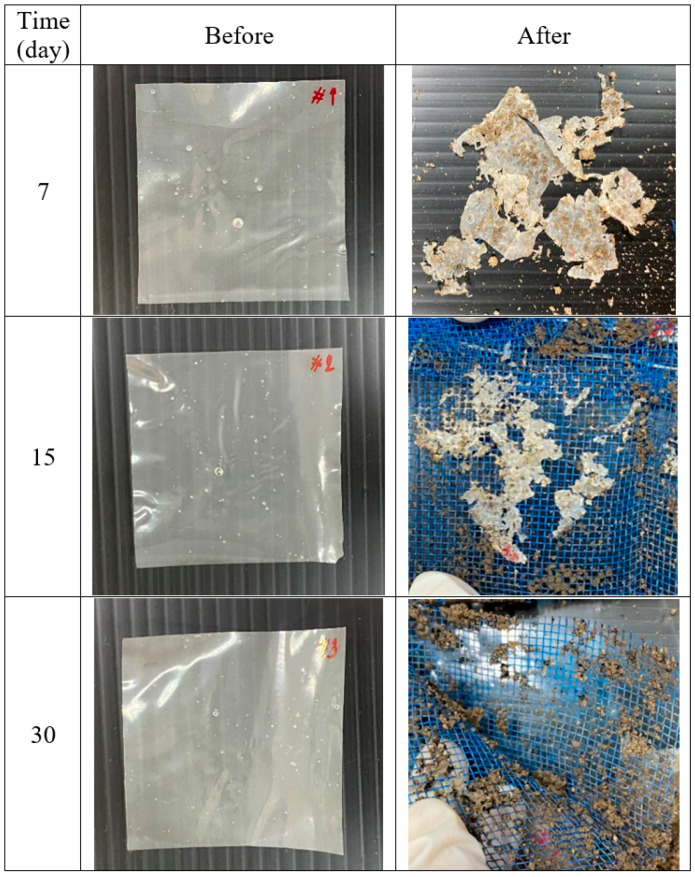
Photographs of G25CA15 specimens before and after being buried in the soil for different periods of time.

All film samples clearly deteriorated in the burial test. All films broke into large pieces within the first week and became smaller as time progressed. The film completely disintegrated after 30 days. No difference can be observed between films containing different amounts of citric acid, unlike other crosslinked starch systems which take longer to degrade [42]. Photographs of the samples before and after the soil burial test are shown in Figure 10.

## 4. Discussion

It has been shown that PSS can be easily transformed into useful flexible films. The films do not display surface stickiness and are water-resistant. They also absorb much less water than most unmodified and modified starch films studied so far. The films also have relatively strong mechanical properties. Again, their mechanical strengths are significantly greater than those of most unmodified and modified starch film. The first and foremost reason for these properties to occur is the high amylose content of the starch and its ability to recrystallize upon drying (retrogradation), as evident from X-ray diffraction (Figure 3). The crystalline regions act as a physical crosslink and provide the film with water-resistant property. When glycerol and citric acid were added to the system, the crystallinity of the films was not much affected and yet the modulus and strength of the film decreased. Thus, it could be deduced that both glycerol and citric acid stay in and plasticize the amorphous region [41,60], leading to softer materials with a greater elongation at the break. Citric acid also acts as a crosslink agent via the formation of ester linkages between starch molecules [39,59]. Furthermore, citric acid aids in heat sealing of the film and its higher citric acid content provides greater seal strength.

PSS films can be prepared without requiring any chemical treatment or modification, resulting in low associated material and energy intensities. Additionally, all the ingredients are biobased, and the films can completely biodegrade in the natural environment in just a few weeks. Considering these facts, PSS film is a good choice for single-use packaging. Nevertheless, there are still many aspects of PSS that need to be explored to fully leverage its potential as a material. Greater impact and more applications can be expected once it is more widely studied.

## 5. Conclusions

Water-resistant and biodegradable flexible films were successfully prepared from pineapple stem starch, glycerol, and citric acid. The mechanical properties of the films, i.e., modulus, strength, and elongation at the break, can be adjusted to cover a wide range to suit different intended applications. Films with a greater amount of citric acid have a lower modulus, lower strength, and larger elongation at the break. The films are also heat-sealable, provided that the content of the citric acid is sufficient, allowing them to be conveniently converted into different packaging materials.

## Data Availability

The data presented in this study are available on request from the corresponding author.
